# Decreased Expression of Canstatin in Rat Model of Monocrotaline-Induced Pulmonary Arterial Hypertension: Protective Effect of Canstatin on Right Ventricular Remodeling

**DOI:** 10.3390/ijms21186797

**Published:** 2020-09-16

**Authors:** Akira Sugiyama, Maina Kaisho, Muneyoshi Okada, Kosuke Otani, Hideyuki Yamawaki

**Affiliations:** Laboratory of Veterinary Pharmacology, School of Veterinary Medicine, Kitasato University, Higashi 23 bancho 35-1, Towada city, Aomori 034-8628, Japan; dv17003@st.kitasato-u.ac.jp (A.S.); vm14029g@st.kitasato-u.ac.jp (M.K.); otani@vmas.kitasato-u.ac.jp (K.O.); yamawaki@vmas.kitasato-u.ac.jp (H.Y.)

**Keywords:** canstatin, ELISA, fibrosis, hypertrophy, pulmonary arterial hypertension

## Abstract

Pulmonary arterial hypertension (PAH) is a progressive disease which causes right ventricular (RV) failure. Canstatin, a C-terminal fragment of type IV collagen α2 chain, is expressed in various rat organs. However, the expression level of canstatin in plasma and organs during PAH is still unclear. We aimed to clarify it and further investigated the protective effects of canstatin in a rat model of monocrotaline-induced PAH. Cardiac functions were assessed by echocardiography. Expression levels of canstatin in plasma and organs were evaluated by enzyme-linked immunosorbent assay and Western blotting, respectively. PAH was evaluated by catheterization. RV remodeling was evaluated by histological analyses. Real-time polymerase chain reaction was performed to evaluate RV remodeling-related genes. The plasma concentration of canstatin in PAH rats was decreased, which was correlated with a reduction in acceleration time/ejection time ratio and an increase in RV weight/body weight ratio. The protein expression of canstatin in RV, lung and kidney was decreased in PAH rats. While recombinant canstatin had no effect on PAH, it significantly improved RV remodeling, including hypertrophy and fibrosis, and prevented the increase in RV remodeling-related genes. We demonstrated that plasma canstatin is decreased in PAH rats and that administration of canstatin exerts cardioprotective effects.

## 1. Introduction

Pulmonary arterial hypertension (PAH) is a progressive disease characterized by an abnormal elevation of mean PA pressure with PA remodeling [1]. Excessive and chronic pressure overload by PAH leads to right ventricular (RV) remodeling such as hypertrophy and fibrosis. RV failure caused by RV remodeling is the most common complication of PAH [2,3]. Brain natriuretic peptide (BNP) and amino-terminal-proBNP are currently used as a biomarker for risk stratification and monitoring the efficacy of treatment in PAH [4]. Since a multiple biomarker approach could be more effective to evaluate the stage of PAH and its prognosis [4], a novel biomarker is anticipated.

Canstatin, a 24 kDa C-terminal fragment of type IV collagen α2 chain, was originally discovered as a potent endogenous anti-angiogenic factor [5]. To the best of our knowledge, the expression level of canstatin in human organs is currently unknown. We previously demonstrated that canstatin is highly expressed in rat organs including heart and lung [6]. On the other hand, the circulating level of canstatin has not yet been measured either in humans or laboratory animals. We previously demonstrated that expression of canstatin was decreased in the infarcted area of myocardial infarction model rats [6,7]. Thus, the expression level of canstatin may be changed by different cardiovascular diseases. However, it is not clear whether the expression level is altered in PAH.

In the present study, we firstly investigated whether canstatin is a potential novel biomarker for PAH. For this purpose, we measured the plasma concentration of canstatin in a rat model of monocrotaline-induced PAH by using a sandwich enzyme-linked immunosorbent assay (ELISA) established in this study and also evaluated relationships with pathological conditions of PAH. In addition, we assessed protein expression of canstatin in various organs, including the heart, lung, kidney and liver, in PAH rats by Western blotting. We recently demonstrated that long-term administration of recombinant canstatin inhibits cardiac hypertrophy in isoproterenol-injected rats [8]. Thus, in this study, we further investigated whether canstatin administration improves pathological conditions of PAH and RV failure in PAH rats.

## 2. Results

### 2.1. Plasma Concentration of Canstatin Was Decreased in PAH Rats, Which Was Correlated with Pathological Conditions

**Figure 1:**
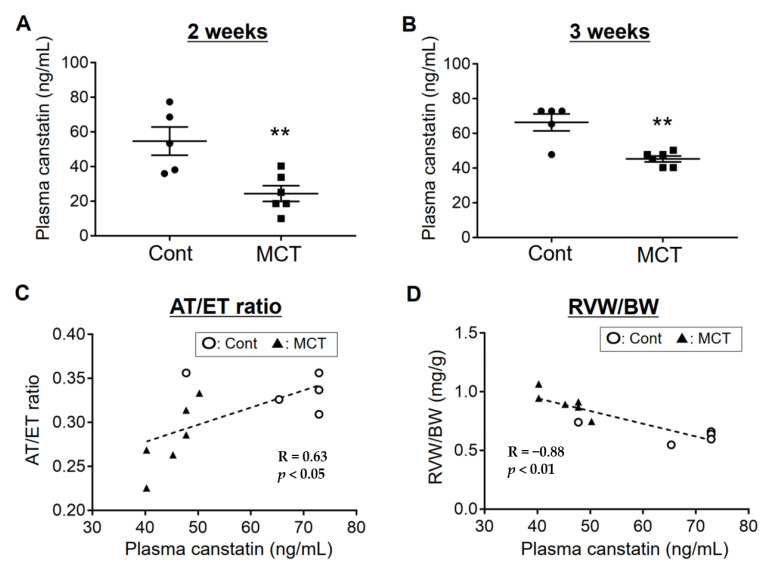
Expression level of canstatin in plasma was decreased and correlated with severity in a rat model of monocrotaline-induced pulmonary arterial hypertension (PAH). Eight-week-old male Wistar rats were intraperitoneally injected with saline (Cont) or monocrotaline (60 mg/kg, MCT). (**A**,**B**) Two (**A**) or three (**B**) weeks later, blood samples were collected and plasma was obtained. Plasma canstatin level was measured by sandwich enzyme-linked immunosorbent assay (ELISA). The concentration of plasma canstatin was shown as scatter plots of individual data points and mean ± standard error of the mean (S.E.M.) (Cont: *n* = 5, MCT: *n* = 6). ** *p* < 0.01 vs. Cont. (**C**,**D**) Relationship between plasma concentration of canstatin and acceleration time (AT)/ejection time (ET) ratio (**C**) or right ventricular (RV) weight (RVW)/body weight (BW) ratio (**D**) was shown as scatter plots of individual data points (Cont: *n* = 5, MCT: *n* = 6). Pearson’s correlation coefficient analysis was performed to evaluate the relationship. R: correlation coefficient.

Eight-week-old male Wistar rats were injected with monocrotaline (60 mg/kg, i.p., MCT) to establish the PAH model, as described previously [9]. Control rats were injected with the same volume of saline (Cont). Three weeks after monocrotaline injection, a decrease in acceleration time (AT)/ejection time (ET) ratio and increase in lung weight (LW) and RV weight (RVW), commonly observed features in a rat model of monocrotaline-induced PAH [10,11], were confirmed (Table 1). The plasma concentration of canstatin in PAH rats (2 weeks: 24.4 ± 4.5 ng/mL, 3 weeks 45.3 ± 1.7 ng/mL) was significantly decreased compared with Cont (2 weeks: 54.7 ± 8.2 ng/mL, 3 weeks: 66.4 ± 4.9 ng/mL) (*p* < 0.01, Figure 1A,B). In addition, we evaluated the correlation between plasma concentration of canstatin and pathological conditions of PAH rats. Three weeks after monocrotaline injection, the plasma concentration of canstatin in PAH rats was positively correlated with a reduction in AT/ET ratio (R = 0.63, *p* < 0.05, Figure 1C) and negatively correlated with an increase in RVW/body weight (BW) (R = −0.88, *p* < 0.01, Figure 1D).

### 2.2. Expression of Canstatin in RV, Lung and Kidney Was Decreased in PAH Rats

**Figure 2:**
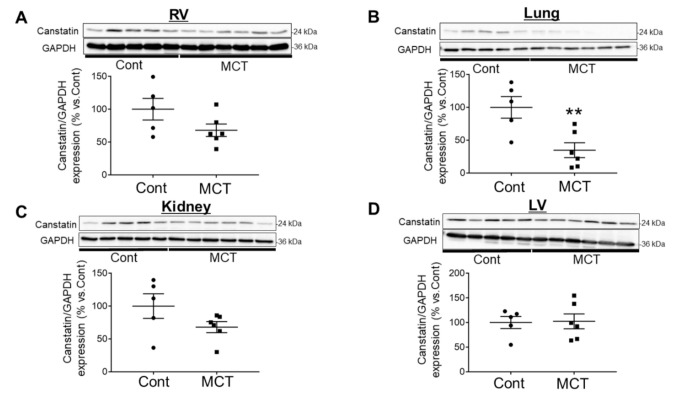
Expression level of canstatin in RV, lung and kidney but not left ventricle (LV) was decreased in a rat model of monocrotaline-induced PAH. Eight-week-old male Wistar rats were intraperitoneally injected with saline (Cont) or monocrotaline (60 mg/kg, MCT). Three weeks later, RV (**A**), lung (**B**), kidney (**C**) and LV (**D**) were isolated and total protein was extracted. The expression of canstatin was measured by Western blotting. (Upper) Representative blots of canstatin and glyceraldehyde 3-phosphate dehydrogenase (GAPDH) are shown. (Lower) Levels of canstatin were corrected by GAPDH, and the normalized expression relative to Cont is shown as mean ± S.E.M. (Cont: *n* = 5, MCT: *n* = 6). ** *p* < 0.01 vs. Cont.

The protein expression of canstatin in PAH rats was decreased in RV (68.0 ± 9.5% vs. Cont) (*p =* 0.11, Figure 2A), lung (34.8 ± 11.3% vs. Cont) (*p <* 0.01, Figure 2B) and kidney (67.9 ± 8.4% vs. Cont) (*p =* 0.13, Figure 2C) but not left ventricle (LV; 102.4 ± 15.1% vs. Cont) (Figure 2D) or liver (113.1 ± 10.1% vs. Cont) (Figure S1).

### 2.3. Canstatin Had No Effect on Monocrotaline-Induced PAH

Next, we examined whether canstatin administration affects monocrotaline-induced PAH. Four-week-old male Wistar rats were divided into the following four groups: vehicle-administered Cont (Cont + vehicle), canstatin-administered Cont (Cont + canstatin), vehicle-administered MCT (MCT + vehicle) and canstatin-administered MCT (MCT + canstatin) (Table 2). 

**Figure 3:**
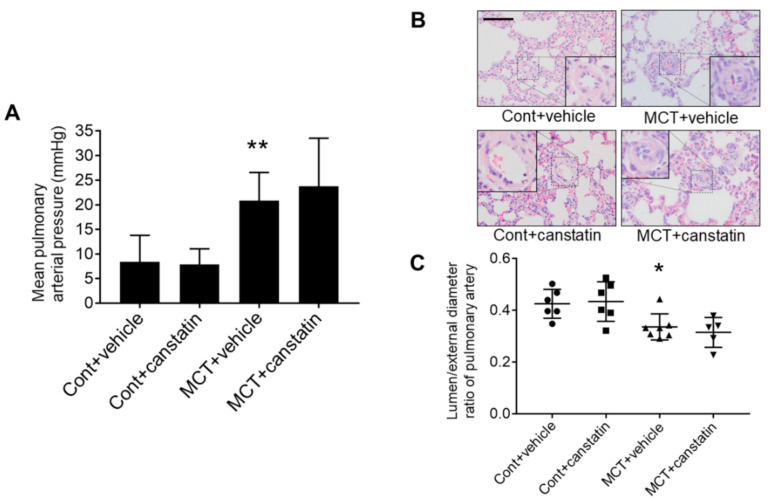
Canstatin had no effect on monocrotaline-induced PAH. Four-week-old male Wistar rats were intraperitoneally injected with saline (Cont) or monocrotaline (60 mg/kg, MCT). Then, vehicle or recombinant mouse canstatin (20 µg/kg) was intraperitoneally administered to the rats for 3 weeks. (**A**) PA pressure was measured by right ventricular catheterization. Mean PA pressure was shown as mean ± S.E.M. (Cont + vehicle: *n* = 7, Cont + canstatin: *n* = 8, MCT + vehicle: *n* = 6, MCT + canstatin: *n* = 5). (**B**,**C**) The lung was isolated and thin paraffin section was made. (**B**) Representative hematoxylin and eosin (HE) stained pictures of the lung from Cont + vehicle, Cont + canstatin, MCT + vehicle and MCT + canstatin are shown. Scale bar: 100 µm. (**C**) Luminal/external diameter ratio (in%) of PA (outer diameter: 30–50 µm) was calculated and is shown as scatter plots of individual data points and mean ± S.E.M. (Cont + vehicle: *n* = 6, Cont + canstatin: *n* = 6, MCT + vehicle: *n* = 7, MCT + canstatin: *n* = 5). *, ** *p* < 0.05, 0.01 vs. Cont + vehicle.

In MCT, AT/ET ratio was significantly decreased (*p* < 0.01 vs. Cont + vehicle), which was not affected by canstatin (Table 2). In MCT, mean PA pressure was significantly increased (*p* < 0.01 vs. Cont + vehicle), which was not affected by canstatin (Figure 3A). In MCT, LW was significantly increased (*p* < 0.01 vs. Cont + vehicle), which was not affected by canstatin (Table 2). In MCT, luminal diameter/external diameter ratio was significantly decreased (*p* < 0.05 vs. Cont + vehicle), which was not affected by canstatin (Figure 3B,C).

### 2.4. Canstatin Improved Monocrotaline-Induced RV Hypertrophy

**Figure 4:**
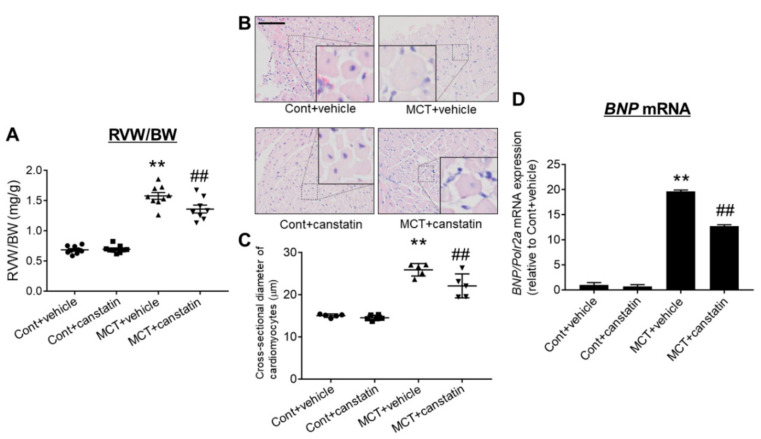
Canstatin improved monocrotaline-induced RV hypertrophy. Four-week-old rats were intraperitoneally injected with saline (Cont) or monocrotaline (60 mg/kg, MCT). Then, vehicle or recombinant mouse canstatin (20 µg/kg) was intraperitoneally administered for 3 weeks. (**A**) RVW/BW was calculated and is shown as scatter plots of individual data points and mean ± S.E.M. (Cont + vehicle: *n* = 9, Cont + canstatin: *n* = 9, MCT + vehicle: *n* = 9, MCT + canstatin: *n* = 8). (**B**,**C**) The RV was isolated and then thin paraffin section was made. (**B**) Representative HE stained pictures of RV from Cont + vehicle, Cont + canstatin, MCT + vehicle and MCT + canstatin are shown. Scale bar: 100 µm. (**C**) Cross-sectional diameter of cardiomyocytes was measured and is shown as scatter plots of individual data points and mean ± S.E.M. (Cont + vehicle: *n* = 4, Cont + canstatin: *n* = 5, MCT + vehicle: *n* = 5, MCT + canstatin: *n* = 5). (**D**) Total RNA was extracted from RV and then complimentary DNA was obtained by reverse transcription. The mRNA expression of *brain natriuretic peptide* (*BNP*) was measured by quantitative real-time polymerase chain reaction (qRT-PCR) (ΔΔCT method). The normalized mRNA expression of *BNP* relative to Cont + vehicle is shown as mean ± S.E.M. (Cont + vehicle: *n* = 8, Cont + canstatin: *n* = 8, MCT + vehicle: *n* = 9, MCT + canstatin: *n* = 8). ** *p* < 0.01 vs. Cont + vehicle, ## *p* < 0.01 vs. MCT + vehicle.

RV hypertrophy and dysfunction are the major comorbidity of PAH [3]. Thus, we next examined the effects of canstatin on RV hypertrophy. In MCT, RVW (*p* < 0.01 vs. Cont + vehicle), RVW/BW ratio (*p* < 0.01 vs. Cont + vehicle) and RVW/LV weight (LVW) ratio (*p* < 0.01 vs. Cont + vehicle) were significantly increased, which were significantly inhibited by canstatin (*p* < 0.01 vs. MCT + vehicle) (Figure 4A, Table 2). In histological experiments, in MCT, cardiomyocyte hypertrophy was significantly induced in RV tissue (*p* < 0.01 vs. Cont + vehicle), which was significantly inhibited by canstatin (*p* < 0.01 vs. MCT + vehicle) (Figure 4B,C). We also examined the effects of canstatin on mRNA expression of hypertrophy-related gene (*BNP*) by quantitative real-time polymerase chain reaction (qRT-PCR). In MCT, mRNA expression of *BNP* was significantly increased (*p* < 0.01 vs. Cont + vehicle), which was significantly inhibited by canstatin (*p* < 0.01 vs. MCT + vehicle) (Figure 4D).

Besides the above, we examined the effects of canstatin on monocrotaline-induced RV dysfunction by measuring tricuspid annular plane systolic excursion (TAPSE), a parameter of RV function, by echocardiography [12]. In MCT, TAPSE was significantly decreased (MCT + vehicle: 0.12 ± 0.00 vs. Cont + vehicle: 0.37 ± 0.01, *p* < 0.01) and canstatin tended to improve it (MCT + canstatin: 0.15 ± 0.01) (Table 2).

### 2.5. Canstatin Improved Monocrotaline-Induced RV Fibrosis

**Figure 5:**
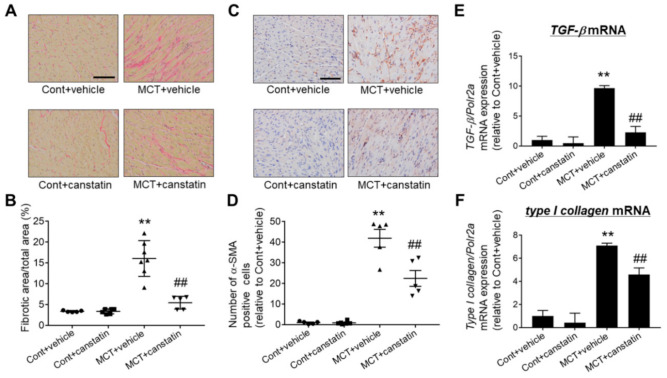
Canstatin improved monocrotaline-induced RV fibrosis. Four-week-old rats were intraperitoneally injected with saline (Cont) or monocrotaline (60 mg/kg, MCT). Then, vehicle or recombinant mouse canstatin (20 µg/kg) was intraperitoneally administered to the rats for 3 weeks. The RV was isolated and then thin paraffin section was made. (**A**) Representative picrosirius red stained pictures of RV from Cont + vehicle, Cont + vehicle, MCT + vehicle and MCT + canstatin are shown. Scale bar: 100 µm. (**B**) Fibrotic area/total area ratio (in%) was measured and is shown as scatter plots of individual data points and mean ± S.E.M. (Cont + vehicle: *n* = 5, Cont + canstatin: *n* = 6, MCT + vehicle: *n* = 7, MCT + canstatin: *n* = 5). (**C**) Representative pictures immunohistochemically stained with a specific antibody against α-smooth muscle actin (α-SMA) in RV from Cont + vehicle, Cont + canstatin, MCT + vehicle and MCT + canstatin are shown. Scale bar: 100 µm. (**D**) The number of α-SMA positive cells in 3 fields was counted and the normalized number relative to Cont + vehicle is shown as scatter plots of individual data points and mean ± S.E.M. (Cont + vehicle: *n* = 5, Cont + canstatin: *n* = 6, MCT + vehicle: *n* = 5, MCT + canstatin: *n* = 5). (**E**,**F**) Total RNA was extracted from RV and then complimentary DNA was obtained by reverse transcription. The mRNA expression of *transforming growth factor-β* (*TGF-β*) (**E**) and *type I collagen* (**F**)) was measured by qRT-PCR (ΔΔCT method). The normalized mRNA expression of *TGF-β* and *type I collagen* relative to Cont + vehicle is shown as mean ± S.E.M. (Cont + vehicle: *n* = 8, Cont + canstatin: *n* = 8, MCT + vehicle: *n* = 9, MCT + canstatin: *n* = 8). ** *p* < 0.01 vs. Cont + vehicle, ## *p* < 0.01 vs. MCT + vehicle.

RV fibrosis is observed in a rat model of monocrotaline-induced PAH [11]. Thus, we examined the effects of canstatin on RV fibrosis by picrosirius red staining. In MCT, the fibrotic area was significantly increased in RV (*p* < 0.01 vs. Cont + vehicle), which was significantly inhibited by canstatin (*p* < 0.01 vs. MCT + vehicle) (Figure 5A,B). Myofibroblasts, characterized by an abundant expression of α-smooth muscle actin (α-SMA), play critical roles during the development of fibrosis in various organs including the heart [13,14]. It was reported that the number of myofibroblasts mainly differentiated from fibroblasts was increased in pathologically fibrotic RV tissue in a rat model of monocrotaline-induced PAH [15]. Thus, we examined the effects of canstatin on the number of myofibroblasts by immunohistochemical staining against α-SMA. In MCT, the number of myofibroblasts was significantly increased in RV (*p* < 0.01 vs. Cont + vehicle), which was significantly inhibited by canstatin (*p* < 0.01 vs. MCT + vehicle) (Figure 5C,D). We also examined the effects of canstatin on mRNA expressions of fibrosis-related genes (*transforming growth factor-β* (*TGF-β*) and *type I collagen*) by qRT-PCR. In MCT, mRNA expression of *TGF-β* and *type I collagen* was significantly increased (*p* < 0.01 vs. Cont + vehicle), which was significantly inhibited by canstatin (*p* < 0.01 vs. MCT + vehicle) (Figure 5E,F).

## 3. Discussion

In the present study, we, for the first time, clarified the expression level of canstatin in plasma and organs of PAH rats, which was significantly correlated with the pathological conditions of PAH. We also demonstrated that canstatin administration attenuated RV remodeling without improving PAH in the rats.

Extracellular matrix-related proteins are one of candidates for a novel biomarker of PAH. Schumann et al. demonstrated that plasma concentration of matrix metalloproteinase-2, tissue inhibitor of matrix metalloproteinase-4 and tenascin C, a matricellular protein, was elevated in the patients with PAH [16]. Serum level of endostatin, a C-terminal fragment of type XVIII collagen α1 chain, was also elevated in the patients with PAH [17]. Although canstatin is widely expressed throughout the body [6], to the best of our knowledge, a circulating concentration of canstatin has not yet been determined. Besides this, it has not been clarified whether the expression level of canstatin is changed in PAH. In the present study, we demonstrated that the plasma concentration of canstatin was 54.7 ± 8.2 ng/mL (2 weeks) and 66.4 ± 4.9 ng/mL (3 weeks) in the control normal rats (Figure 1A,B). A significant decrease in the canstatin concentration was observed in PAH rats (2 weeks: 24.4 ± 4.5 ng/mL, 3 weeks: 45.3 ± 1.7 ng/mL), which coincides with the decreased expression of canstatin in the organs including RV, lung and kidney but not LV and liver (Figure 2 and Figure S1). Interestingly, the plasma concentration of canstatin was correlated with the pathological conditions of PAH (Figure 1C,D). In particular, an increase in RVW/BW (R = −0.88, *p* < 0.01) was much more correlated with the plasma canstatin level than a reduction in AT/ET ratio (R = 0.63, *p* < 0.05). It is thus suggested that canstatin may be a candidate for a novel biomarker for RV hypertrophy in PAH.

We have previously reported that the decrease in canstatin expression in the infarcted area after myocardial infarction in rats was caused possibly by cathepsin S [6]. Chang et al. demonstrated that cathepsin S activity was increased in serum and lung in a rat model of monocrotaline-induced PAH [18]. Acute and chronic kidney diseases are relatively common in PAH patients and the increase in venous pressure by RV dysfunction leads to kidney injury [19]. Steubl et al. demonstrated that the serum level of cathepsin S was increased in mice with a decrease in the glomerular filtration rate [20]. Besides this, a study by Yao et al. demonstrated that the expression of cathepsin S in kidney was increased in a mouse model of hydronephrosis [21]. Therefore, it was presumed that the decreased expression of canstatin was caused by cathepsin S in PAH rats. However, it may be possible that the release of canstatin from type IV collagen α2 chain was decreased. Therefore, further study is needed to clarify the detailed mechanism of the downregulation of canstatin.

Next, we investigated whether canstatin administration improves the pathological conditions of PAH. However, canstatin administration had no effect on PA pressure and lumen stenosis of PA in PAH rats (Figure 3). The anti-angiogenic effect of canstatin on cancer model mice was observed in the range of 3–10 mg/kg [5,22], which is much higher than the dose used in this study (20 µg/kg). Significant intimal hyperplasia via abnormal proliferation of endothelial cells was observed in a rat model of Sugen 5419/hypoxia-induced but not monocrotaline-induced PAH [23]. Further examinations are needed in a future study to explore whether a higher dose of canstatin improves PAH in the Sugen 5419/hypoxia model.

On the other hand, canstatin administration significantly inhibited monocrotaline-induced hypertrophy, fibrosis and upregulation of the responsible genes in RV (Figure 4 and Figure 5). We previously reported that canstatin suppressed isoproterenol-induced cardiac hypertrophy via inhibiting Ca^2+^/calcineurin/nuclear factor of activated T cells (NFAT) pathway [8]. RV hypertrophy in PAH was induced by pressure overload, which caused a rise in intercellular Ca^2+^ concentration ([Ca^2+^]_i_) in cardiomyocytes [2,24]. The increase in [Ca^2+^]_i_ activated the calcineurin/NFAT pathway and promoted the transcription of hypertrophic genes including *BNP* [25]. Besides this, Martínez-Martínez et al. reported that cardiac-specific calcineurin deletion markedly reduced angiotensin II-induced cardiac fibrosis and mRNA expression of the *TGF-β* family in mice [26]. From these observations, it is suggested that canstatin might inhibit monocrotaline-induced RV hypertrophy and fibrosis in part through the suppression of the Ca^2+^/calcineurin/NFAT pathway. To clarify the detailed mechanism by which canstatin inhibits monocrotaline-induced RV remodeling, further experiments are required.

## 4. Materials and Methods

### 4.1. Regents and Antibodies

Reagent sources: Monocrotaline (Wako, Osaka, Japan) and recombinant mouse canstatin (produced by our laboratory [8]).

Antibody sources: anti-α-SMA antibody (Dako, Glostrup, Denmark), anti-canstatin antibody (Boster Biological Technology, Pleasanton, CA, USA), anti-glyceraldehyde 3-phosphate dehydrogenase (GAPDH) antibody (Wako), anti-rabbit IgG horseradish peroxidase whole antibody and anti-mouse IgG horseradish peroxidase (Cell Signaling Technology, Danvers, MA, USA).

### 4.2. Animal Study

Animal studies approved by the Institutional Animal Care and Use Committee of the School of Veterinary Medicine, the Kitasato University (Approval No. 18-060 (18 June 2018), 19-014 (19 June 2019), 19-179 (10 February 2020)) were carried out in accordance with the institutional guidelines of the Kitasato University.

Experiments for measurement of canstatin expression level in PAH rats: Eight-week-old male Wistar rats (Clea Japan, Tokyo, Japan) were injected with monocrotaline (60 mg/kg, i.p., MCT) to establish the PAH model, as described previously [9]. Control rats were injected with same volume of saline (Cont). Two or three weeks later, blood samples were drawn from saphenous vein under isoflurane anesthesia (2–3%) and collected in microtubes containing ethylenediaminetetraacetic acid (EDTA). Plasma samples were obtained by centrifuging the blood samples at 1200 g for 15 min at 4 °C and preserving them at −80 °C. Following the blood sampling at 3 weeks, echocardiography was performed under isoflurane anesthesia (2–3%). Then, the rats were deeply anesthetized with urethane (1.5 g/kg, i.p.; Sigma Aldrich, St. Louis, MO, USA) and the hearts, lungs, kidneys and livers were isolated.

Experiments for effects of recombinant canstatin on PAH rats: Four-week-old male Wistar rats (Clea Japan) were divided into the following 4 groups: vehicle-administered Cont (Cont + vehicle), canstatin-administered Cont (Cont + canstatin), vehicle-administered MCT (MCT + vehicle) and canstatin-administered MCT (MCT + canstatin). Recombinant canstatin (20 µg/kg) or vehicle (0.8 mM Tris, 20 mM L-arginine, 4% glycerol in saline) was intraperitoneally administered once a day for 21 days from the day of a single injection of monocrotaline (60 mg/kg, i.p.) or saline. Three weeks later, echocardiography and measurement of PA pressure by catheterization were performed. Following the measurements, the hearts and lungs were isolated under deep urethane anesthesia (1.5 g/kg, i.p.). After 3 weeks following the monocrotaline injection, all rats in Cont survived (Cont + vehicle and Cont + canstatin: 100%; 9/9). On the other hand, the survival rate of MCT + vehicle group was decreased (81.8%; 9/11), which was not affected by canstatin (MCT + canstatin: 88.9%; 8/9).

### 4.3. Measurement of Plasma Canstatin by Sandwich ELISA

We established a sandwich ELISA for canstatin (Supplementary Materials [8,27], Figure S2, Table S1) and the plasma concentration of canstatin was measured. Briefly, the absorbance at 450 and 560 nm of plasma samples diluted at 1:100 in Plasma Sample Diluent (ImmunoChemistry Technologies, Bloomington, MN, USA) was measured by using a TriStar LB941 multimode microplate reader (Berthold, Bad Wildbad, Germany), and the subtracted values [(readings at 450 nm)–(reading at 560 nm)] were calculated. Then, the plasma concentration of canstatin was determined by a regression formula of standard curve created with recombinant mouse canstatin (0.391, 0.781, 1.563, 3.125, 6.25, 12.5 and 25 ng/mL).

### 4.4. Echocardiography

Echocardiography was performed under isoflurane anesthesia (2–3%) using SonoScape X5V (SonoScape Medical Corp., Shenzhen, China) as described previously [9]. AT and ET of PA flow were measured by pulse doppler mode. TAPSE was obtained by M-mode from apical four chamber views.

### 4.5. Western Blotting

To measure protein expression of canstatin in organs including RV, lung, kidney, LV and liver, Western blotting was performed [6]. Total proteins were extracted by RIPA buffer (20 mM Tris-HCl pH 7.4, NaCl 150 mM, MgCl_2_ 10 mM, 1% TritonX-100, 0.1% sodium dodecyl sulfate, 0.5% sodium deoxycholate) containing 1% protease inhibitor cocktail (Nacalai Tesque, Kyoto, Japan). Equal amount of proteins was separated by sodium dodecyl sulfate–polyacrylamide gel electrophoresis and transferred to a nitrocellulose membrane. After being blocked with 0.5% skim milk, the membranes were incubated with primary antibody against canstatin (1:500 dilution) or GAPDH (1:2000 dilution) overnight at 4 °C. After the reaction with a horseradish peroxidase-conjugated secondary antibody and EZ-ECL detecting reagent (Biological Industries, Kibbutz Beit Haemek, Israel), the visualized proteins were detected using an ATTO light capture system (AE-6972; ATTO, Tokyo, Japan) and analyzed by using a CS Analyzer software (Version 3.0, ATTO).

### 4.6. Measurement of PA Pressure

Mean PA pressure was measured by right ventricular catheterization [28]. A catheter connected to a BP transducer (MLT0670; AD Instruments, Colorado Springs, CO, USA) was inserted into the PA from right external jugular vein in rats under urethane anesthesia (1.5 g/kg, i.p.). Mean PA pressure was digitally recorded using the BP Amp (ML117; AD Instruments) and PowerLab 2/25 system (ML825; AD Instruments).

### 4.7. Histological Analysis

The formalin-fixed RV and lung tissues were embedded in paraffin and thin sections (4 µm) were made. Hematoxylin and eosin (HE) staining and picrosirius red staining were performed as described previously [29]. The images were obtained using a light microscope (BX-51; OLYMPUS, Tokyo, Japan) equipped with a microscope digital camera (DP74; OLYMPUS). Luminal and external diameter of 3 PA (diameter: 50–100 µm) from each lung section stained with HE was measured by cellSens Imagine Software (Version 1.17, OLYMPUS), and luminal diameter/external diameter ratio (in%) was calculated. Diameter of 50 cardiomyocytes from each RV section stained with HE was measured by cellSens Imagine Software (Version 1.17). Fibrotic area of 3 high power fields from each RV section stained with picrosirius red was measured by Image J software (Version 1.52a, NIH, Bethesda, MD, USA), and fibrotic area/total area (in%) was calculated.

Immunohistochemical staining was performed with avidin-biotin complex method [30]. The deparaffinized sections were heated in 10 mM sodium citrate buffer (pH 6.0) by a microwave and incubated in methanol with 3% H_2_O_2_. Then, the sections were blocked with 5% normal goat serum and incubated with primary antibody against α-SMA (1:100 dilution) overnight at 4 °C. After the sections were incubated in biotinylated link (Dako) and then in streptavidin-horseradish peroxidase (Dako) at room temperature, anti-α-SMA antibody was visualized by a liquid 3,3’-Diaminobenzidine (DAB) + substrate chromogen system (Dako). The images were obtained using a light microscope (BX-51) equipped with a microscope digital camera (DP74). The number of non-vascular α-SMA positive cells in 3 fields from each RV section was counted.

### 4.8. qRT-PCR

To measure mRNA expression of hypertrophy and fibrosis-related genes in RV, qRT-PCR was performed [8]. The frozen RV were homogenized with cell destroyer (Bio Medical Science Inc., Tokyo, Japan), and total RNA was extracted by TRI regent (Molecular Research Center, Inc., Cincinnati, OH, USA). cDNA was synthesized from 1 µg of total RNA using the ReverTra Ace qPCR master mix (Toyobo, Osaka, Japan). The PCR amplification was performed using the Thunderbird SYBR qPCR mix (Toyobo) with PikoRealTM Real-Time PCR System (Thermo Fisher Scientific, Waltham, MA, USA). The mRNA expression level of rat *BNP*, *TGF-β* and *type I collagen* was measured by ΔΔCT method [relative to *RNA polymerase II subunit A* (*Polr2a*)]. The primer sequences are shown in Table S2.

### 4.9. Statistical Analysis

The results are presented as mean ± standard error of the mean (S.E.M.). In two-group comparison, statistical analyses were performed by unpaired two-tailed Student’s *t* test (Figure 1A,B and Figure 2, Table 1). In multi-group comparison, statistical analyses were performed by one-way ANOVA followed by Bonferroni’s post-hoc test (Figure 3, Figure 4 and Figure 5, Table 2). Pearson’s correlation coefficient analysis was performed to evaluate relationships between plasma concentration of canstatin and pathological conditions of PAH (Figure 1C,D). A value of *p* < 0.05 was considered statistically significant.

## 5. Conclusions

Our study for the first time demonstrated that plasma concentration of canstatin is downregulated with the decrease of expression level in RV, lung and kidney of monocrotaline-induced PAH rats, which was correlated with the pathological conditions of PAH. We further demonstrated that canstatin improves pathological RV remodeling.

## Figures and Tables

**Table 1:** Body weight (BW), acceleration time (AT)/ejection time (ET) ratio, lung weight (LW) and right ventricular (RV) weight (RVW) of rats used to measure expression levels of canstatin in plasma and tissues.

	Cont	MCT
BW (g)	339 ± 8	287 ± 4 **
AT/ET ratio	0.34 ± 0.01	0.28 ± 0.02 **
LW (mg)	1157 ± 36	1576 ± 64 **
RVW (mg)	217 ± 14	260 ± 13 **
RVW/BW ratio (mg/g)	0.64 ± 0.03	0.90 ± 0.04 **

Cont: saline-injected rats; MCT: monocrotaline-injected rats. The data are shown as mean ± standard error of the mean (S.E.M.) (Cont: *n* = 5, MCT: *n* = 6). ** *p* < 0.01 vs. Cont (unpaired two-tailed Student’s *t* test).

**Table 2:** BW, echocardiographic parameters, tissue weights and survival rate of rats used to investigate effects of canstatin administration on MCT.

	Cont + Vehicle	Cont + Canstatin	MCT + Vehicle	MCT + Canstatin
BW (g)	241 ± 4	240 ± 5	186 ± 4 **	195 ± 6
AT/ET ratio	0.38 ± 0.01	0.40 ± 0.01	0.22 ± 0.01 **	0.23 ± 0.01
TAPSE	0.37 ± 0.01	0.39 ± 0.02	0.12 ± 0.00 **	0.15 ± 0.01
(LV + S)W (mg)	608 ± 14	596 ± 9	524 ± 10 **	577 ± 18 #
RVW (mg)	164 ± 4	167 ± 6	293 ± 11 **	263 ± 11
RVW/BW ratio (mg/g)	0.68 ± 0.02	0.69 ± 0.02	1.58 ± 0.06 **	1.36 ± 0.07 ##
RVW/(LV + S)W ratio (mg/mg)	0.27 ± 0.01	0.28 ± 0.01	0.56 ± 0.02 **	0.46 ± 0.01 ##
LW (mg)	1033 ± 18	1013 ± 19	1424 ± 58 **	1491 ± 74

Cont + vehicle: vehicle-administered Cont, Cont + canstatin: canstatin-administered Cont, MCT + vehicle: vehicle-administered MCT, MCT + canstatin: canstatin-administered MCT, TAPSE: tricuspid annular plane systolic excursion, (LV + S)W: left ventricular plus interventricular septum weight. The data are shown as mean ± S.E.M. (Cont + vehicle: *n* = 9, Cont + canstatin: *n* = 9, MCT + vehicle: *n* = 9, MCT + canstatin: *n* = 8). ** *p* < 0.01 vs. Cont + vehicle; #, ## *p* < 0.05, 0.01 vs. MCT + vehicle (one-way ANOVA followed by Bonferroni’s post-hoc test).

## Supplementary Materials

Supplementary materials can be found at https://www.mdpi.com/1422-0067/21/18/6797/s1.

## Abbreviations

PAH	pulmonary arterial hypertension
RV	right ventricle
BNP	brain natriuretic peptide
ELISA	enzyme-linked immunosorbent assay
MCT	monocrotaline-injected rats
Cont	saline-injected rats
AT	acceleration time
ET	ejection time
LW	lung weight
RVW	RV weight
BW	body weight
LV	left ventricle
LVW	LV weight
qRT-PCR	quantitative real-time polymerase chain reaction
TAPSE	tricuspid annular plane systolic excursion
α-SMA	α-smooth muscle actin
TGF-β	transforming growth factor-β
NFAT	nuclear factor of activated T cells
GAPDH	glyceraldehyde 3-phosphate dehydrogenase
EDTA	ethylenediaminetetraacetic acid
HE	hematoxylin and eosin
DAB	diaminobenzidine
Polr2a	RNA polymerase II subunit A
S.E.M.	standard error of the mean
