# Supplementary material for: Decreased Expression of Canstatin in Rat Model of Monocrotaline-Induced Pulmonary Arterial Hypertension: Protective Effect of Canstatin on Right Ventricular Remodeling
Source: Int J Mol Sci. 2020 Sep 16;21(18):6797. doi: 10.3390/ijms21186797 (PMC7554857; doi:10.3390/ijms21186797)
Supplement: Supplementary file 1 [file ijms-21-06797-s001.pdf]

Decreased expression of canstatin in rat model of monocrotaline-induced pulmonary arterial hypertension: protective effect of canstatin on right ventricular remodeling

Akira Sugiyama[1], Maina Kaisho[1], Muneyoshi Okada[1]*, Kosuke Otani[1] and Hideyuki Yamawaki[1]

[1] Laboratory of Veterinary Pharmacology, School of Veterinary Medicine, Kitasato University, Higashi 23 bancho 35-1, Towada city, Aomori 034-8628, Japan; dv17003@st.kitasato-u.ac.jp (A.S.); vm14029g@st.kitasato-u.ac.jp (M.K.); otani@vmas.kitasato-u.ac.jp (K.O.); yamawaki@vmas.kitasato-u.ac.jp (H.Y.)

*CORRESPONDENCE TO: Muneyoshi Okada, DVM, PhD.

Laboratory of Veterinary Pharmacology,

School of Veterinary Medicine, Kitasato University, Higashi 23 bancho 35-1, Towada city, Aomori 034-8628, Japan.

Phone: +81-176-23-4371; FAX: +81-176-23-8703

e-mail: mokada@vmas.kitasato-u.ac.jp

Supplementary Materials

Methods

Production and purification of monoclonal anti-canstatin antibodies

The mouse monoclonal antibody (mAb) against mouse canstatin was constructed by the mouse medial iliac lymph node method [1]. Briefly, recombinant mouse canstatin produced in our laboratory [2] was injected into the tail base of mice. Lymphocytes from the medial iliac lymph node of the immunized mice were corrected and fused with mouse myeloma. The hybridoma cells were screened and three hybridoma cells producing monoclonal antibodies against canstatin (mAb 9-10F, mAb 10-8D and mAb 23-4B) were obtained. The mAbs were purified from serum-free hybridoma media (Wako, Osaka, Japan) by using a spin column based antibody purification kit (Protein G) (Cosmo Bio, Tokyo, Japan) according to the manufacturer protocol. The purification of mAbs was confirmed by sodium dodecyl sulfate-polyacrylamide gel electrophoresis (SDS-PAGE) following coomassie brilliant blue staining (**Supplementary Figure. 2A**) and Western blotting (**Supplementary Figure. 2B**).

Establishment of sandwich enzyme-linked immunosorbent assay (ELISA) for canstatin

Both mAb 9-10F and mAb 10-8D were used as a capture antibody. The mAb 23-4B labeled with biotin by using Biotin Labeling Kit-NH_2 (DOJINDO, Kumamoto, Japan) according to the

manufacturer protocol was used as a detection antibody. Each well of the 96-well microtiter plate was coated with 100 μL of mAb 9-10F and mAb 10-8D diluted in carbonate/bicarbonate buffer (pH 9.6) (1.5 μg/mL each, total concentration of mAb: 3 μg/mL) overnight at 4 ºC. After washing each well with phosphate buffered saline (PBS) containing 0.05% Tween-20 (PBS-T), the plate was blocked with 200 μL of blocking buffer [0.5% bovine serum albumin (BSA) in PBS] for 2 h at room temperature. After washing with PBS-T, 100 μL of plasma sample diluted at 1:100 in Plasma Sample Diluent (ImmunoChemistry Technologies, Bloomington, MN, USA) or recombinant mouse canstatin diluted in the same diluent as a standard was added and incubated for 2 h at 37 ºC. After washing with PBS-T, 100 μL of biotin-conjugate mAb 23-4F diluted at 0.5 μg/mL in blocking buffer was added and incubated for 2 h at room temperature. After washing with PBS-T, 100 μL of horse radish peroxidase-conjugated streptavidin (Proteintech, Rosemont, IL, USA) diluted at 1:2500 in blocking buffer was added and incubated for 30 min at room temperature. After washing with PBS-T, 100 μL of a peroxidase substrate, TMB solution (Nacalai Tesque, Kyoto, Japan) was added to each well and incubated for 30 min at room temperature under shading. The enzymatic reaction was stopped by adding 100 μL of 2% H_2SO_4. The absorbance at 450 nm and 560 nm was measured by using a TriStar LB941 multimode microplate reader (Berthold, Bad Wildbad, Germany) and subtracted value [(reading at 450 nm)–(reading at 560 nm)] was calculated. Standard curve for concentration of recombinant canstatin with 2-fold serial dilution (0.391, 0.781, 1.563, 3.125, 6.25, 12.5 and 25 ng/mL) vs. absorbance was created (**Supplementary Figure. 2C**). The plasma concentration of canstatin was

calculated from the standard curve. The accuracy of this assay was confirmed by performing a spike and recovery test, which showed 99.9% of recovery rate (**Supplementary Table 1**).

Reference

1. Sado, Y.; Inoue, S.; Tomono, Y.; Omori, H. Lymphocytes from enlarged iliac lymph nodes as fusion partners for the production of monoclonal antibodies after a single tail base immunization attempt. *Acta Histochem. Cytochem.* **2006**, *39*, 89–94, doi:10.1267/ahc.06001.
2. Sugiyama, A.; Okada, M.; Yamawaki, H. Canstatin suppresses isoproterenol-induced cardiac hypertrophy through inhibition of calcineurin/nuclear factor of activated T-cells pathway in rats. *Eur. J. Pharmacol.* **2020**, *871*, 172849, doi:10.1016/j.ejphar.2019.172849.

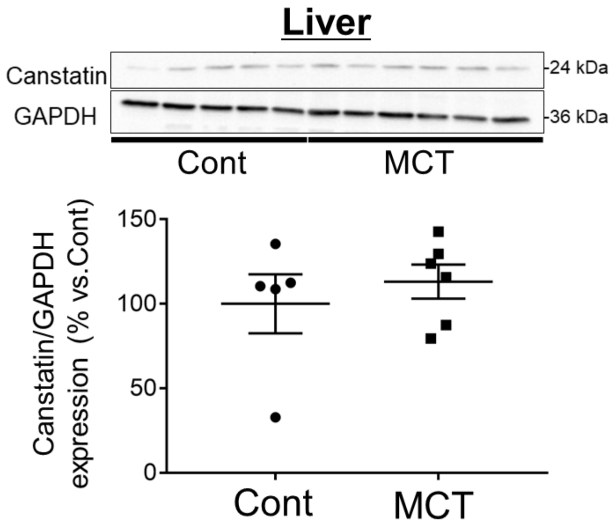

Supplementary Figure 1. Expression level of canstatin in liver

Eight-weeks-old male Wistar rats were intraperitoneally injected with saline (Cont) or monocrotaline (60 mg/kg, MCT). Three weeks later, liver was isolated and total protein was extracted. The expression of canstatin was measured by Western blotting. (Upper) Representative blots of canstatin and glyceraldehyde 3-phosphate dehydrogenase (GAPDH) were shown. (Lower) Levels of canstatin were corrected by GAPDH, and the normalized expression relative to Cont was shown as mean±standard error of the mean (S.E.M.) (Cont: n=5, MCT: n=6).

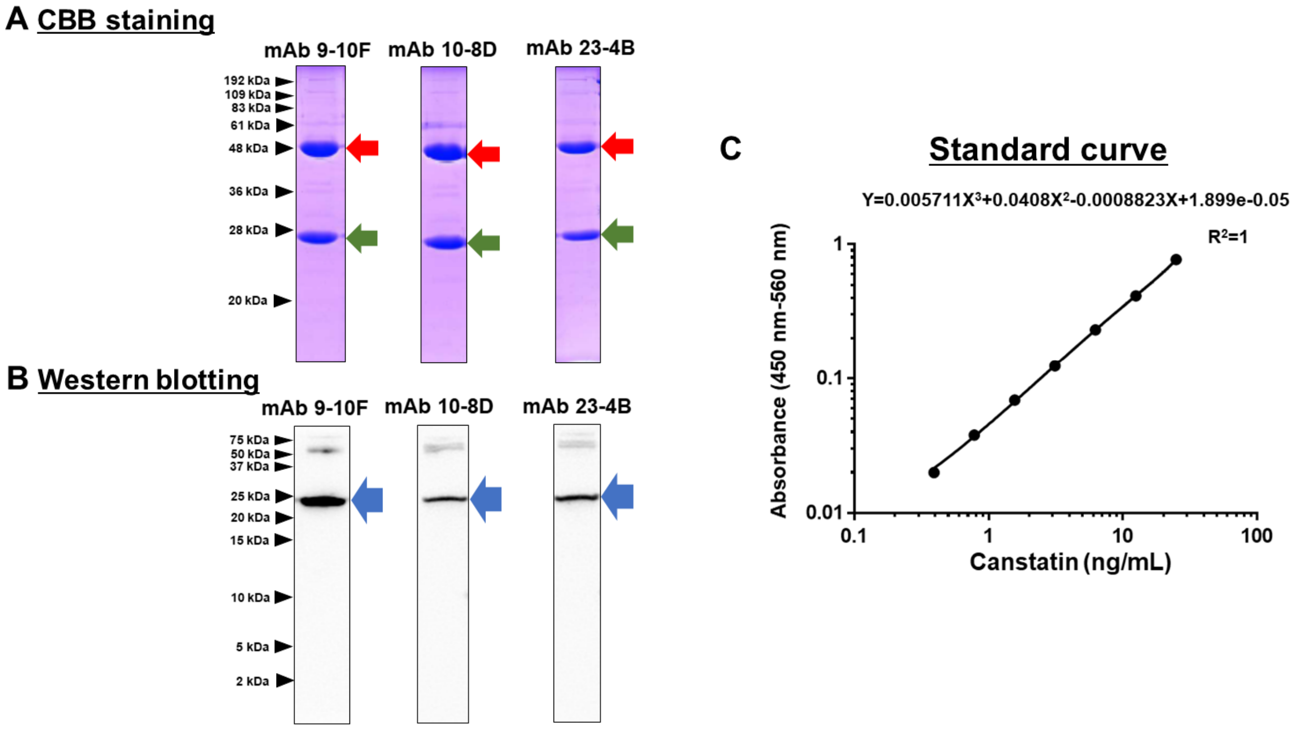

Supplementary Figure 2. Purification of monoclonal anti-canstatin antibodies and standard curve for concentration of canstatin vs. absorbance by sandwich enzyme-linked immunosorbent assay (ELISA).

(A) Representative coomassie brilliant blue (CBB) staining images for monoclonal anti-canstatin antibodies (mAbs; mAb 9-10F, mAb 10-8D and mAb 23-4B) after sodium dodecyl sulfate-polyacrylamide gel electrophoresis were shown. The bands were detected at predicted size for heavy chain (≈50 kDa; Red arrows) and light chain (≈25 kDa; Green arrows) of IgG. (B) The reactivity of mAbs against recombinant mouse canstatin was confirmed by Western blotting. Representative blots probed with anti-canstatin mAbs were shown. The bands were detected at predicted size of canstatin (≈24 kDa; Blue arrows). (C) Representative standard curve for concentration of canstatin with 2-fold serial dilution (0.391, 0.781, 1.563, 3.125, 6.25, 12.5 and 25 ng/mL) vs. absorbance (450 nm-560 nm) was shown.

	Spiked diluent	Spiked plasma sample	Unspiked plasma sample	Recovery (%)
Spike (ng/mL)	Measured concentration (ng/mL) (A)	Measured concentration (ng/mL) (B)	Measured concentration (ng/mL) (C)	(B-C)/A × 100
0.781	0.753	1.592	0.840	99.9

Supplementary Table 1. Spike and recovery test for accuracy of canstatin sandwich enzyme-linked immunosorbent assay (ELISA)

Spike and recovery test was performed to evaluate accuracy of the established canstatin sandwich ELISA assay. The standard protein (recombinant mouse canstatin: 0.781 ng/mL) was added (spiked) into diluent (A) or rat plasma sample (dilution at 1:100) (B). Absorbance of spiked diluent (A), spiked plasma sample (B) and unspiked plasma sample (C) was measured and the concentration (ng/mL) of canstatin was calculated from standard curve. The recovery (%) was calculated as follows: [(B) measured concentration of spiked plasma sample (ng/mL) - (C) measured concentration of unspiked plasma sample (ng/mL)]/(A) measured concentration of spiked diluent (ng/mL)×100.

Genes	Accession number	Primer sequence (5’-3’)
rat *BNP*	NM_031545	Forward: GAACAATCCACGATGCAGAAGC<br>Reverse: GGGCCTTGGTCCTTTGAGAG
Rat *TGF-β*	NM_021578	Forward: GACCGCAACAACGCAATCTA<br>Reverse: TTCCGTCTCCTTGGTTCAGC
Rat *type I collagen*	NM_053304	Forward: GGATCGACCCTAACCAAGGC<br>Reverse: GATCGGAACCTTCGCTTCCA
Rat *Polr2a*	XM_343922	Forward: CGTATCCGCATCATGAACAGTGA<br>Reverse: TCATCCATCTTATCCACCACCTCTT

Supplementary Table 2. Sequences of primers for quantitative real-time PCR

BNP: *brain natriuretic peptide*; *TGF-β*: *transforming growth factor-β*; *Polr2a*: *RNA polymerase II subunit A*
